# Association Between Leisure Activity Patterns and Cognitive Function for U.S. Older Adults

**DOI:** 10.1093/geroni/igaf122.2555

**Published:** 2025-12-31

**Authors:** Yan-Jhu Su, Elizabeth Dugan, Jaqueline Avila, Laura Hayman

**Affiliations:** The Pennsylvania State University, University Park, Pennsylvania, United States; University of Massachusetts Boston, Boston, Massachusetts, United States; University of Massachusetts Boston, Boston, Massachusetts, United States; University of Massachusetts Boston Manninng College of Nursing & Health Sciences, Boston, Massachusetts, United States

## Abstract

This study explores the longitudinal impact of leisure activity patterns on cognitive function among U.S. older adults, utilizing data from the Health and Retirement Study (HRS) and the Consumption and Activities Mail Survey (CAMS). Using sequence analysis and clustering, five distinct leisure activity patterns were identified, ranging from diverse engagement to restricted or declining participation. Participants in the “All Activities” cluster demonstrated the highest cognitive scores, while those in “Gradual Cessation of Activities” or “Household-Only Activities” exhibited significant cognitive decline. Stratified analyses revealed that older adults, women, and individuals with lower educational attainment were more adversely affected by limited or inconsistent activity patterns. The findings highlight the protective role of diverse and sustained leisure engagement in cognitive health and underscore the moderating effects of demographic factors, including age, gender, and education. These results emphasize the need for tailored interventions to promote diverse leisure activities, address demographic disparities, and support healthy cognitive aging in diverse populations.

